# Comparison of Cardiovascular Outcomes Between Dapagliflozin and Empagliflozin in Patients With Type 2 Diabetes: A Meta-Analysis

**DOI:** 10.7759/cureus.27277

**Published:** 2022-07-26

**Authors:** Rahat A Memon, Hanieh Akbariromani, Rimsha R Vohra, Hayan Kundi, Rao Faraz Saleem, Muhammad abuzar Ghaffari, Donald Haas, Areeba Khan

**Affiliations:** 1 Internal Medicine, Abington Memorial Hospital, Abington, USA; 2 Medical School, Islamic Azad University, Tehran, IRN; 3 Internal Medicine, Dow Medical College, Karachi, PAK; 4 Medical School, Fazaia Medical College, Karachi, PAK; 5 Internal Medicine, Akhtar Saeed Medical and Dental College, Lahore, PAK; 6 Anatomy, Akhtar Saeed Medical and Dental College, Lahore, PAK; 7 Cardiovascular Medicine, Abington Memorial Hospital, Abington, USA; 8 Critical Care Medicine, United Medical and Dental College, Karachi, PAK

**Keywords:** type 2 diabetes, comparison, cardiovascular outcomes, empaglifozin, dapaglifozin

## Abstract

Sodium-glucose cotransporter-2 (SGLT2) inhibitors are oral diabetes medications that enhance the excretion of glucose by preventing the renal proximal tubules from reabsorbing glucose, which lowers glucose levels in plasma. Currently, studies have shown that SGLT2 inhibitors have beneficial impacts on cardiovascular outcomes, but their effect varies between the individual SGLT2 inhibitors. Thus, the current meta-analysis was conducted to compare the efficacy of dapagliflozin and empagliflozin in preventing cardiovascular events in patients with type 2 diabetes. The current meta-analysis was conducted using the Preferred Reporting Items for Systematic Reviews and Meta-Analyses (PRISMA) reporting guidelines. A search of studies comparing cardiovascular events between dapagliflozin and empagliflozin in patients with type 2 diabetes published up to 1 July 2022 was done by two reviewers independently on PubMed, Embase and Cumulated Index to Nursing and Allied Health Literature (CINAHIL). The pre-specified primary endpoints were cardiovascular death, stroke, myocardial infarction and heart failure. Overall four studies were included in this meta-analysis. No significant difference was found in the incidence of myocardial infarction (risk ratio (RR)=0.81, 95% confidence interval (CI): 0.60-1.09), heart failure (RR=0.76, 95% CI: 0.56-1.04), cardiovascular mortality (RR=0.46, 95% CI: 0.18-1.20) and stroke (RR=1.07, 95% CI: 0.84-1.38) between dapagliflozin and empagliflozin. Results have shown that the risk of developing stroke, heart failure, myocardial infarction and cardiovascular death were not significantly different in the two groups.

## Introduction and background

Oral diabetic drugs known as sodium-glucose cotransporter-2 (SGLT2) inhibitors increase the excretion of glucose by preventing glucose from being reabsorbed by the renal proximal tubules, which reduces plasma glucose levels [1]. Several randomized control trials have shown that SGLT2 inhibitors can enhance cardiovascular outcomes in diabetic patients [1-2]. Type 2 diabetes is one of the major risk factors for microvascular and macrovascular diseases [3]. Nowadays, the treatment for diabetes has broadened from glycemic control to a more patient-centered approach with consideration of the hazard of heart failure and atherosclerotic cardiovascular disease [4]. This approach is based on a new drug like SGLT2 inhibitors and its impact on heart failure prevention has attracted unparalleled interest. The SGLT2 inhibitor is currently regarded as a critical medication for diabetes mellitus from the perspective of preventing future cardiovascular disease (CVD) events as a result of the accumulating clinical evidence [5].

SGLT2 inhibitors along with glycemic control have a positive impact on certain cardiometabolic markers like uric acid, blood pressure, and body weight [6] and thus the rate of prescription of SGLT2 inhibitors has increased for patients with diabetes mellitus. For instance, in the United States, the percentage of type 2 diabetes mellitus patients prescribed SGLT2 inhibitors increased to 11.9% in 2019 from 3.8% in 2015 [7]. However, not every SGLT2 inhibitors share the same pharmacokinetic properties, for instance, dapagliflozin's longer-lasting pharmacological effects last even 18 hours after administration, whereas empagliflozin effects start to noticeably diminish 12 hours after administration [8]. Because dapagliflozin's sodium diuresis and sodium excretion effects last longer and are more stable than those of empagliflozin, it has been found to reduce the 24-hour fluctuation in blood pressure. Thus, it is linked to a lower risk for cardiovascular diseases [9].

Currently, studies have shown that SGLT2 inhibitors have beneficial impacts on cardiovascular outcomes, but their effect varies between the individual SGLT2 inhibitors. Thus, the current meta-analysis has been conducted to compare the efficacy of dapagliflozin and empagliflozin in preventing cardiovascular events in patients with type 2 diabetes.

## Review

Methodology

The current meta-analysis was conducted using the Preferred Reporting Items for Systematic Reviews and Meta-Analyses (PRISMA) reporting guidelines. A search of studies comparing cardiovascular events between dapagliflozin and empagliflozin in patients with type 2 diabetes published up to 1 July 2022 was done by two reviewers independently on PubMed, Embase and CINAHIL without restrictions on language and publication date. The key terms used were “dapagliflozin”, “empagliflozin”, “cardiovascular events” and “type 2 diabetes” including their synonyms and subheadings. Studies were eligible if they compared the cardiovascular events between dapagliflozin and empagliflozin in patients with type 2 diabetes. Studies were excluded if they compared either of these two medications with a placebo or any other SGLT2 inhibitor drug. Besides this, studies were also excluded if they did not report any cardiovascular outcomes such as cardiovascular death, stroke, myocardial infarction and heart failure. Discrepancy over eligibility was resolved through discussion or consensus with a third reviewer. Study characteristics such as author name, year of publication, outcomes assessed, inclusion criteria and follow-up time were extracted and presented in the form of a table.

Study Endpoints

For this meta-analysis, the pre-specified primary endpoints were cardiovascular death, stroke, myocardial infarction and heart failure. Most inclusive definitions were used as reported in the original article including ancillary papers or online supplementary materials. Two reviewers independently extract endpoint tallies into a structured dataset.

Risk of Bias Assessment

To assess the quality of included studies, Newcastle-Ottawa Scale (NOS) was used. This scale is based on three major components included assessment of outcomes, adjustment for potential confounding variables and selection of study patients. Each study may receive up to Nine points based on this scale. Articles with a NOS score of five or higher were regarded as high-quality publications in the current study [10]. In the current meta-analysis, all studies were retrospective, so the rate of loss to follow-up was not assessed, therefore, each study was assessed on a scale of eight points. 

Data Synthesis and Data Analysis

The pooled relative risk for each of the primary outcomes was calculated by applying the Mantel-Haenszel method combined with a fixed effect model along with their 95% confidence interval (CI). P-values were considered significant at a level of <0.05. Heterogeneity of treatment effect among studies was assessed by computing the I^2^ index. If the I^2^ index was less than 25%, heterogeneity was considered to be low, moderate if it was between 25% and 70%, and high if it was more than 75%. In the current meta-analysis, we were not able to assess publication bias because of the low number of included studies. Data analysis was done using Revman Review Manager v. 5.4.1 (The Cochrane Collaboration, London, UK).

Results

**Figure 1:**
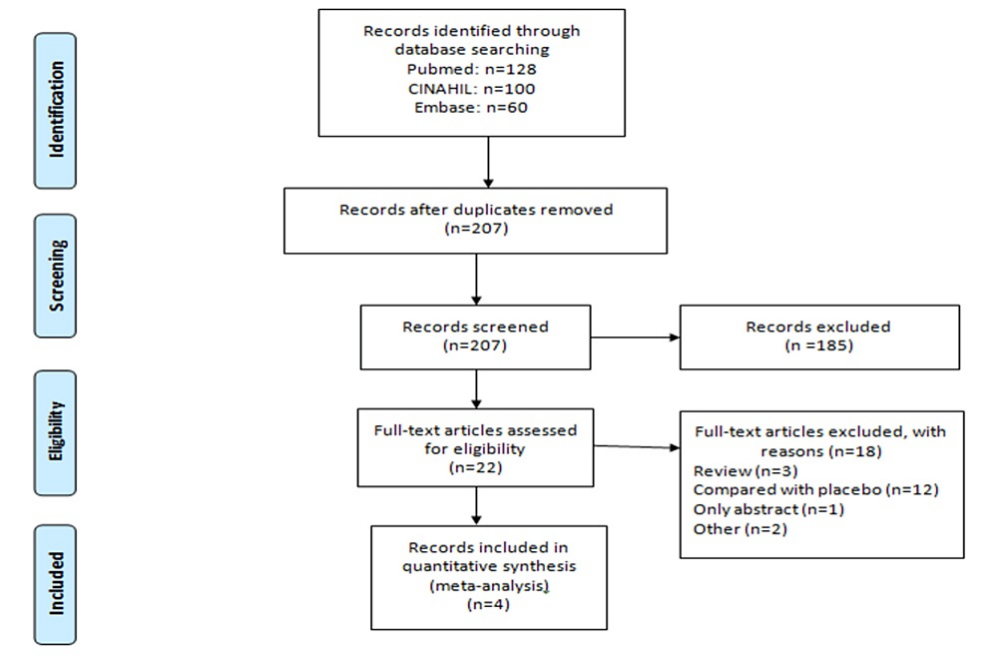
Flowchart of the study selection

We included the studies from inception to 1 July 2022 and obtained 288 articles. After removing duplicates, the title and abstracts of 207 articles were screened based on inclusion criteria. Overall, 22 articles were eligible for full-text screening, and finally, four studies were included in this meta-analysis [11-14]. The PRISMA flow chart is shown in Figure 1. The main characteristics of all included studies have been shown in Table 1. All four studies were retrospective and involved a total of 25715 patients with type 2 diabetes (12644 in the Dapagliflozin group and 13071 in the Empagliflozin group). The mean follow-up period of each of the included studies was more than two years as shown in Table 1.

**Table 1:** Characteristics of included studies

First Author	Year of Publication	Study Type	Groups	Sample Size	Population	Follow-up Period
Lim et al. [11]	2022	Retrospective	Dapagliflozin	921	Patients with type 2 diabetes prescribed empagliflozin, dapagliflozin	55.1 months
			Empagliflozin	921		
Park et al. [12]	2022	Retrospective	Dapagliflozin	609	Patients with DM who treated SGLT2 inhibitors such as DAPA or EMPA	38.9 months
			Empagliflozin	600		
Shao et al. [13]	2019	Retrospective	Dapagliflozin	5812	Type 2 diabetes patients newly treated with an SGLT2 inhibitor, either dapagliflozin or empagliflozin	32.66 months
			Empagliflozin	6869		
Suzuki et al. [14]	2022	Retrospective	Dapagliflozin	5302	Patients with DM who were treated with SGLT2 inhibitors	29.13 months
			Empagliflozin	4681		

Risk of Bias

The details of the risk of bias assessment of the included publications are shown in Table 2. All studies included in the meta-analysis had high quality. Two studies scored 7 [12-13], while two studies scored 8 points out of a total score of 8 [11,14].

**Table 2:** Risk of bias assessment of the included studies

First author	Representativeness of exposed cohort	Selection of nonexposed cohort	Ascertainment of exposure	Demonstration that outcome of interest was not present at the start of the study	Adjusting for the most important risk factors	Adjusting for other risk factors	Assessment of outcome	Follow-up length	Total score
Lim et al. 2022 [11]	1	1	1	1	1	1	1	1	8
Park et al. 2022 [12]	1	1	1	1	0	1	1	1	7
Shao et al. 2019 [13]	1	1	0	1	1	1	1	1	7
Suzuki et al. 2022 [14]	1	1	1	1	1	1	1	1	8

Comparison of Cardiovascular Outcomes Between Dapagliflozin and Empagliflozin

**Figure 2:**
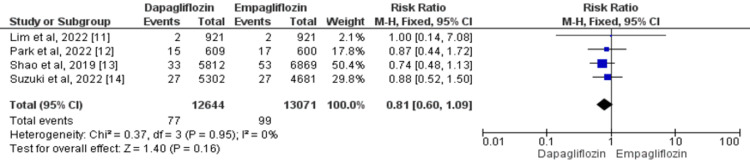
Forest plots for meta-analysis of the effects of dapagliflozin vs. empagliflozin on myocardial infarction Each square and horizontal line denotes the point estimate and 95% CI for each trial’s RR. The diamonds signify the pooled RR; the diamond’s center denotes the point estimate and the width denotes the 95% CI. CI: confidence interval; RR: risk ratio Sources: References 11-14 [11-14]

Overall, four studies compared the risk of myocardial infarction between two groups [11-14]. In patients with type 2 diabetes, the risk of MI was not significantly different between patients who received dapagliflozin and patients who received empagliflozin (risk ratio (RR)=0.81, 95% confidence interval (CI): 0.60-1.09). No heterogeneity of treatment effect among studies was there (I^2^=0%) as shown in Figure 2.

**Figure 3:**
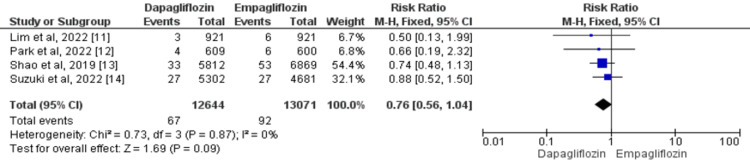
Forest plots for meta-analysis of the effects of dapagliflozin vs. empagliflozin on heart failure. Each square and horizontal line denotes the point estimate and 95% CI for each trial’s RR. The diamonds signify the pooled RR; the diamond’s center denotes the point estimate and the width denotes the 95% CI. CI: confidence interval; RR: risk ratio Sources: References 11-14 [11-14]

Four studies were analyzed to assess the effects of dapagliflozin and empagliflozin on the risk of heart failure in patients with type 2 diabetes [11-14]. No significant difference in risk of heart failure was there between dapagliflozin and empagliflozin (RR=0.76, 95% CI: 0.56-1.04). There was no substantial heterogeneity across studies (I^2^=0%) as shown in Figure 3.

**Figure 4:**
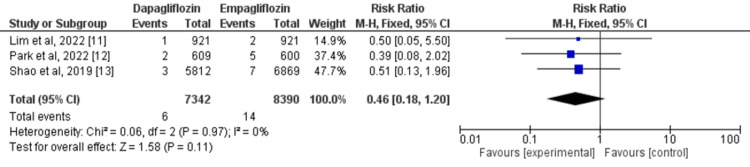
Forest plots for meta-analysis of the effects of dapagliflozin vs. empagliflozin on cardiovascular mortality. Each square and horizontal line denotes the point estimate and 95% CI for each trial’s RR. The diamonds signify the pooled RR; the diamond’s center denotes the point estimate and the width denotes the 95% CI. CI: confidence interval; RR: risk ratio Sources: References 11-13 [11-13]

Three studies were employed to compare the effects of dapagliflozin and empagliflozin on the risk of cardiovascular mortality [11-13]. No significant difference was found in the risk of cardiovascular mortality in the dapagliflozin group compared with the empagliflozin group in patients with type 2 diabetes (RR=0.46, 95% CI: 0.18-1.20). There was no substantial heterogeneity across studies (I^2^=0%) as shown in Figure 4.

**Figure 5:**
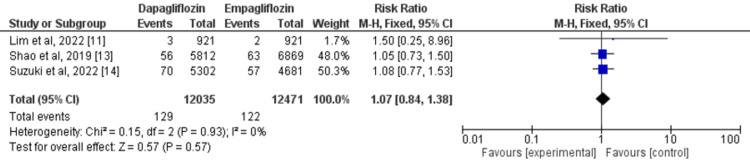
Forest plots for meta-analysis of the effects of dapagliflozin vs. empagliflozin on stroke. Each square and horizontal line denotes the point estimate and 95% CI for each trial’s RR. The diamonds signify the pooled RR; the diamond’s center denotes the point estimate and the width denotes the 95% CI. CI: confidence interval; RR: risk ratio Sources: References 11,13-14 [11,13-14]

Three studies were employed to compare the effects of dapagliflozin and empagliflozin on the risk of stroke [11, 13-14]. The risk of stroke was not significantly different in type 2 diabetic patients who received dapagliflozin and who received empagliflozin (RR=1.07, 95% CI: 0.84-1.38). There was no substantial heterogeneity across studies (I^2^=0%) as shown in Figure 5.

Discussion

In the current meta-analysis of four studies with 25715 patients including 12644 in the Dapagliflozin group and 13071 in the Empagliflozin group, we compared the efficacy of dapagliflozin and empagliflozin in preventing cardiovascular outcomes in patients with type 2 diabetes. The previous meta-analysis has shown that SGLT2 inhibitors were associated with a reduction in cardiovascular deaths and myocardial infarction compared with placebo [15]. However, the effect of different SGLT2 inhibitors varies among the individual SGLT2 inhibitors, this meta-analysis chose only those studies comparing dapagliflozin and empagliflozin to assess which of these two drugs are more effective in preventing cardiovascular outcomes such as myocardial infarction, stroke, heart failure and cardiac-related death in type 2 diabetes patients. The current meta-analysis has shown that no significant difference was there in the risk of developing heart failure, myocardial infarction, stroke, and cardiac death among patients taking empagliflozin and dapagliflozin. It is interesting that there was no concrete proof that specific drug kinds in this class have distinct impacts on cardiovascular outcomes or mortality. Because SGLT2 inhibitors have the same mechanism of action, it is possible that specific medications in this class, such as dapagliflozin, and empagliflozin, have comparable functional effects on cardiovascular events [16]. To the best of our knowledge, this is the first meta-analysis conducted to compare the wide range of cardiovascular outcomes in patients with type 2 diabetes treated with dapagliflozin and empagliflozin.

Diabetes mellitus increases the risk of different cardiovascular outcomes such as atrial fibrillation, coronary artery disease, heart failure, and cardiovascular deaths [17-18] and thus, clinical trials show the robust cardiovascular benefits of SGLT2 inhibitors for patients with diabetes mellitus, revolutionized clinical practice [1]. However, different studies have shown that the effects of SGLT2i may vary according to the type of drug. Shao et al. conducted a multi-institutional study and found that dapagliflozin may offer more favorable benefits in relation to the prevention of heart failure as compared to empagliflozin [13]. The more strong impacts of dapagliflozin over empagliflozin for heart failure can be explained by SGLT2 and SGLT1 receptor selectivity ratio that is lower for dapagliflozin as compared to empagliflozin [19].

More specifically, cardiac ischemia and hypertrophy are related to SGLT1 overexpression in the myocardium, where SGLT2 receptors are never expressed. According to this result, the SGLT2i with a lesser specificity for SGLT2 receptors and a stronger impact on SGLT1 has an even more advantageous impact on HF prevention [11]. Besides this, dapagliflozin did not enhance plasma noradrenaline and aldosterone levels compared to empagliflozin, which could be beneficial for the prevention of heart failure [20]. Based on current recommendations, SGLTS inhibitors need to be considered as a second-line treatment after metformin in type 2 diabetes patients [21]. The recommended starting dose of dapagliflozin is 5 mg once daily and the dosage can be increased to 10 mg once daily in patients who require additional glycemic control [22]. Empagliflozin is available in 10 mg and 25 mg tablets, with a recommended initial dose of 10 mg daily [23].

The current meta-analysis has certain limitations. Firstly, only four studies were included in this paper and all these four studies were performed in a retrospective manner. Secondly, we were not able to assess publication bias as the number of studies was small. Thirdly, none of the included studies mentioned the dosage of drugs that were given to patients. Thus, future studies need to be conducted to compare the impacts of dapagliflozin and empagliflozin in a prospective trial or using studies from multiple centers.

## Conclusions

In summary, the current meta-analysis compared the effects of dapagliflozin and empagliflozin on cardiovascular outcomes in patients with type 2 diabetes. Results have shown that the risks of developing stroke, heart failure, myocardial infarction, and cardiac-related death are not different in the two groups. It is suggested that SGLT2 inhibitors can reduce the risk of cardiovascular diseases but the current study did not identify any significant difference in the effectiveness of dapagliflozin and empagliflozin. In the future, more prospective studies need to be carried out to identify which of these two drugs are more effective in preventing cardiovascular outcomes in patients with type 2 diabetes.
